# Decoding Post-Viral Fatigue: The Basal Ganglia’s Complex Role in Long-COVID

**DOI:** 10.3390/neurolint16020028

**Published:** 2024-03-28

**Authors:** Thorsten Rudroff

**Affiliations:** 1 Department of Health and Human Physiology, University of Iowa, Iowa City, IA 52242, USA; thorsten-rudroff@uiowa.edu; Tel.: +1-(319)-467-0363; Fax: +1-(319)-355-6669; 2 Department of Neurology, University of Iowa Hospitals and Clinics, Iowa City, IA 52242, USA

**Keywords:** basal ganglia, long-COVID, fatigue, neuroimaging

## Abstract

Long-COVID afflicts millions with relentless fatigue, disrupting daily life. The objective of this narrative review is to synthesize current evidence on the role of the basal ganglia in long-COVID fatigue, discuss potential mechanisms, and highlight promising therapeutic interventions. A comprehensive literature search was conducted using PubMed, Scopus, and Web of Science databases. Mounting evidence from PET, MRI, and functional connectivity data reveals basal ganglia disturbances in long-COVID exhaustion, including inflammation, metabolic disruption, volume changes, and network alterations focused on striatal dopamine circuitry regulating motivation. Theories suggest inflammation-induced signaling disturbances could impede effort/reward valuation, disrupt cortical–subcortical motivational pathways, or diminish excitatory input to arousal centers, attenuating drive initiation. Recent therapeutic pilots targeting basal ganglia abnormalities show provisional efficacy. However, heterogeneous outcomes, inconsistent metrics, and perceived versus objective fatigue discrepancies temper insights. Despite the growing research, gaps remain in understanding the precise pathways linking basal ganglia dysfunction to fatigue and validating treatment efficacy. Further research is needed to advance understanding of the basal ganglia’s contribution to long-COVID neurological sequelae and offer hope for improving function across the expanding affected population.

## 1. Introduction

Long-COVID is defined as the continuation or development of new symptoms 3 months after the initial SARS-CoV-2 infection, with these symptoms lasting for at least 2 months with no other explanation [1]. Neuropsychiatric manifestations are common, with severe fatigue reported in upwards of 80% of patients [2,3]. This fatigue is unrelenting, profoundly limits daily functioning, and lacks effective treatments so far. Understanding the underpinnings of long-COVID fatigue is critical, given projections that long-COVID could affect over 100 million people globally [4].

**Figure 1:**
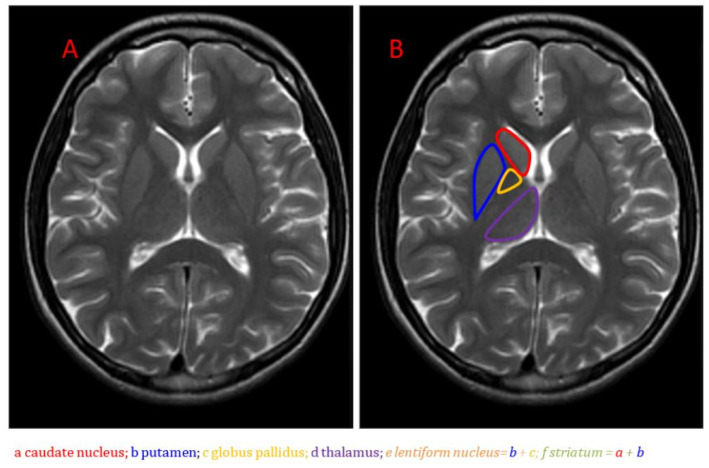
Anatomical MRI of the basal ganglia in the axial plane on T2-weighted imaging: (**A**) Unlabeled image; (**B**) Image with caudate nucleus, putamen, globus pallidus, and thalamus labeled [7].

Recently, attention has turned to the basal ganglia—an interconnected group of subcortical nuclei known to regulate sleep–wake drive, motivation, motor control, and effort-based decision making [5]. Comprised of structures, including the striatum, pallidum, substantia nigra, and subthalamic nucleus, the basal ganglia receive input from and signal to numerous cortical areas [6] (Figure 1; [7]). This strategic placement at the intersection of the limbic, cognitive, and motor loops suggests the basal ganglia may orchestrate diverse aspects of behavior and physiology relevant to fatigue.

Notably, emerging neuroimaging evidence reveals that long-COVID patients exhibit inflammation, hypometabolism, and disrupted connectivity patterns involving basal ganglia circuits [8,9,10,11]. The specificity of the subcortical changes points to dysfunctional signaling between the basal ganglia and interconnected networks as a driver of ongoing symptoms. Understanding exactly how basal ganglia pathology influences cortical excitation and motivation deficits could shed light on post-viral fatigue mechanisms. It may also uncover tailored therapeutic opportunities. Clarifying the basal ganglia’s role in the “long-COVID fog” is critical, given the scale of the long-COVID crisis.

The objective of this narrative review is to synthesize current evidence on the role of the basal ganglia in long-COVID fatigue, discuss potential mechanisms, and highlight promising therapeutic interventions.

To gather relevant literature for this narrative review, a comprehensive search was conducted using PubMed, Scopus, and Web of Science databases. The search included combinations of keywords such as ‘long-COVID’, ‘fatigue’, ‘basal ganglia’, ‘neuroimaging’, ‘inflammation’, ‘dopamine’, and ‘treatment’. The search was limited to English articles published between January 2020 and December 2023. Reference lists of relevant articles were also manually screened for additional studies. The inclusion criteria focused on studies investigating neurological symptoms, particularly fatigue, in long-COVID patients, using neuroimaging techniques to examine brain changes, exploring the role of the basal ganglia, and discussing potential treatments targeting basal ganglia dysfunction.

## 2. Basal Ganglia Dysfunction in Long-COVID Fatigue: Evidence from Neuroimaging

Emerging data from structural and functional neuroimaging studies reveals COVID-19 survivors exhibit specific abnormalities localized within subcortical basal ganglia networks. Inflammation, hypometabolism, and disrupted connectivity involving the caudate, putamen, pallidum, and nucleus accumbens point to the basal ganglia as a driver of persistent neurological symptoms.

### 2.1. Evidence of Metabolic Dysfunction

Quantitative MRI techniques allow inference of chemical environment signatures within the brain. Lu et al. [12] utilized magnetic resonance spectroscopy (MRS) to uncover aberrant metabolic patterns in striatal regions among patients with 10 months long-COVID infection compared to controls. Specifically, altered choline and lactate suggested ongoing neuroinflammation, while reduced glutamate intimated possible interference in excitatory signaling. Further, critically lowered N-acetylaspartate hinted at compromised neuronal integrity. Together, these metabolic perturbations centered around the caudate and putamen nuclei propose that long-COVID disrupts basal ganglia homeostasis. Another study [13] that examined brain MRIs in long-COVID patients found that 56% of them had basal ganglia hyperintensities, suggesting brain tissue damage and inflammation. These MRI abnormalities within the basal ganglia could be indicative of neuroinflammation, hypoperfusion, and other pathological processes affecting deep brain structures in patients with lingering effects from COVID-19. Another study by Kandemirli et al. [14] also noted T1 hyperintensities in the basal ganglia of long-COVID patients. Additionally, magnetic resonance spectroscopy (MRS) showed reduced N-acetylaspartate (NAA)/creatine (Cr) and NAA/choline (Cho) ratios, implying neuronal loss and decreased energy production. These metabolic changes were associated with neurological symptoms like headache, vertigo, and cognitive problems.

Further MRI studies demonstrate focal inflammation concentrated inside the basal ganglia in patients with lingering issues like fatigue and cognitive impairment in long-COVID patients [3,10,15]. This inflammation correlates with clinical measures of brain fog and exertion intolerance. PET imaging similarly shows aberrant hypometabolism clustered around striatal and pallidal regions [16]. The imaging signature shares a resemblance to that seen in autoimmune disorders like lupus and Still’s disease and could reflect localized neuropathology.

A recent FDG-PET pilot study by Luo et al. [17] demonstrated that both fatigued and non-fatigued long-COVID patients showed reduced glucose hypometabolism in the globus pallidus (GP) region of the basal ganglia compared to healthy controls. Specifically, this suggests factors beyond just fatigue influencing basal ganglia dysfunction. The non-fatigued group showed even greater GP hypometabolism compared to the fatigued group. Multiple factors could explain this relative dissociation—patients without fatigue may have greater baseline GP activity that has declined less from pre-infection levels. Alternatively, more severe GP neuroinflammation in non-fatigued patients could be impairing function through alternative pathways besides energy depletion. This also indicates additional pathways contributing to motivational and cognitive deficits distinct from subjective fatigue. A hemisphere effect was observed with greater right GP hypometabolism in patients regardless of fatigue status. This laterality suggests potential asymmetric impacts on information processing between the basal ganglia hemispheres. The basal ganglia, especially the right GP, appears susceptible to various insults from COVID-19 infection. The reasons underlying this regional vulnerability require further study, but inflammation, hypoxia, and anxiety are possibilities. Most critically, dysfunction within this subcortical motivational circuitry could help explain the diversity of neurocognitive symptoms many patients face during long-COVID convalescence.

Table 1 concisely organizes the key findings related to basal ganglia changes and their associations with long-COVID fatigue and other persistent symptoms across multiple studies with varying patient populations and follow-up timepoints. The structural and functional alterations consistently point to a subcortical substrate for lingering neurological issues.

### 2.2. Functional Connectivity Disruptions

Moving beyond static chemical shifts, resting-state functional MRI delineates activity relationships between disparate brain areas. Douaud et al. [10] mapped functional connectivity in over 600 individuals previously infected with SARS-CoV-2. Recovered participants demonstrated consistent connectivity weakening between basal ganglia, thalami, and primary sensory regions versus controls. Similar patterns emerged in an independent cohort of long haulers, corroborating this distinct signature of dopaminergic circuitry dysregulation. The authors suggest long-term fatigue and cognitive and mood symptoms may arise from disrupted communication between striatal dopamine pathways and upstream sensory input.

Equally striking are the functional MRI and SPECT experiments revealing deficient connectivity selectively involving basal ganglia pathways [18,19]. Reduced coherence within nigrostriatal and corticostriatal networks agrees with complaints of motivation loss and mental fatigue. A failure of the basal ganglia relay centers to modulate signals between limbic motivational inputs and prefrontal cognitive controllers may manifest behaviorally as exhaustion. Treatments restoring basal ganglia communication could improve energy levels by reintegrating mood and movement processes.

Similarly, another fMRI study by Huang et al. [16] revealed decreased connectivity between the globus pallidus and frontoparietal executive control networks. The extent of globus pallidus decoupling was proportional to the severity of cognitive impairment with inattention and memory retrieval issues. Disrupted basal ganglia–frontoparietal connectivity likely contributes to executive dysfunction commonly reported by long-COVID patients. In addition to functional connectivity disruptions, molecular PET imaging provides evidence of dopaminergic abnormalities stemming from the basal ganglia in long-COVID.

### 2.3. Mismatch in Structural Architecture

Finally, structural neuroimaging indicates anatomical rearrangements accompanying the functional abnormalities described above. Using multi-contrast MRI, a 2022 study [10] uncovered relative volume expansions in the caudate and accumbens regions and constriction within the globus pallidi among long-COVID subjects. Enlargement in the dorsal striatum areas may reflect inflammation, microgliosis, or vasogenic dysfunction, while pallidal atrophy hints at neurodegeneration. Such structural asymmetry again intimates that long-COVID may perturb basal ganglia nuclei balances. Patients additionally demonstrated reduced cortical surface area and disruptions to thalamic radiations, which together support subcortical involvement. The studies on basal ganglia dysfunction in long-COVID patients provide some clues about neurological mechanisms that may contribute to chronic fatigue, but more research is needed to confirm the links. Another study utilizing diffusion tensor imaging (DTI) by Zhou et al. [20] revealed altered structural connectivity between basal ganglia components. Patients displayed reduced white matter integrity in nigrostriatal pathways. However, they exhibited increased structural connectivity in ventromedial limbic and frontoparietal circuits. This mismatch implies a complex reorganization of structural networks involving the basal ganglia early on in the disease.

Hafiz et al. [21] examined gray matter volume (GMV) differences between 46 recovering COVID-19 patients and 30 healthy controls, as well as correlations between GMV and self-reported fatigue levels. MRI scans were conducted 2 weeks after hospital discharge. The COVID-19 group showed significantly higher fatigue levels than controls. They also showed significantly higher GMV in multiple limbic system regions (e.g., hippocampus, amygdala) and basal ganglia regions (e.g., putamen, pallidum). Across both groups, higher fatigue levels positively correlated with GMV in the posterior cingulate cortex, precuneus, and superior parietal lobe. However, the COVID-19 group showed significantly stronger positive correlations between fatigue levels and GMV in these regions compared to controls. The GMV differences align with findings from single-patient case studies showing neurological involvement in acute COVID-19 infection. The correlation between higher GMV and more fatigue also fits with existing research linking these brain regions to fatigue in other disorders. The authors conclude that even 2 weeks after discharge, recovering COVID-19 patients show GMV alterations in regions related to acute symptoms, as well as a stronger link between GMV in fatigue-related areas and self-reported fatigue levels. This may provide insight into persistent neurological symptoms among COVID-19 survivors.

Heine et al. [22] examined structural brain changes associated with long-COVID fatigue in 50 patients compared to 47 healthy controls, as well as 47 multiple sclerosis (MS) patients with fatigue. At a median of 7.5 months after acute COVID-19, 47 patients showed moderate–severe fatigue. These long-COVID patients also showed higher levels of anxiety, depression, daytime sleepiness, and sleep problems compared to controls. MRI analyses revealed structural changes in the thalamus and basal ganglia of long-COVID patients, including volume loss, surface deformations, and altered diffusion parameters. These subcortical changes correlated with fatigue severity and everyday impairment. Long-COVID patients showed overlapping but less extensive subcortical changes compared to MS patients. In MS, changes were more related to overall lesion burden rather than fatigue. Long-COVID fatigue severity was associated with sleep quality and depression but not acute COVID-19 severity or duration. The authors conclude that the persistent fatigue in long-COVID syndrome has a distinct structural substrate focused on the thalamus and basal ganglia. This provides insight into the neurological impact of long-COVID. In summary, this study demonstrates structural brain changes linked to long-term long-COVID fatigue, focused on subcortical regions that align with fatigue-related changes seen in other disorders like MS.

**Figure 2:**
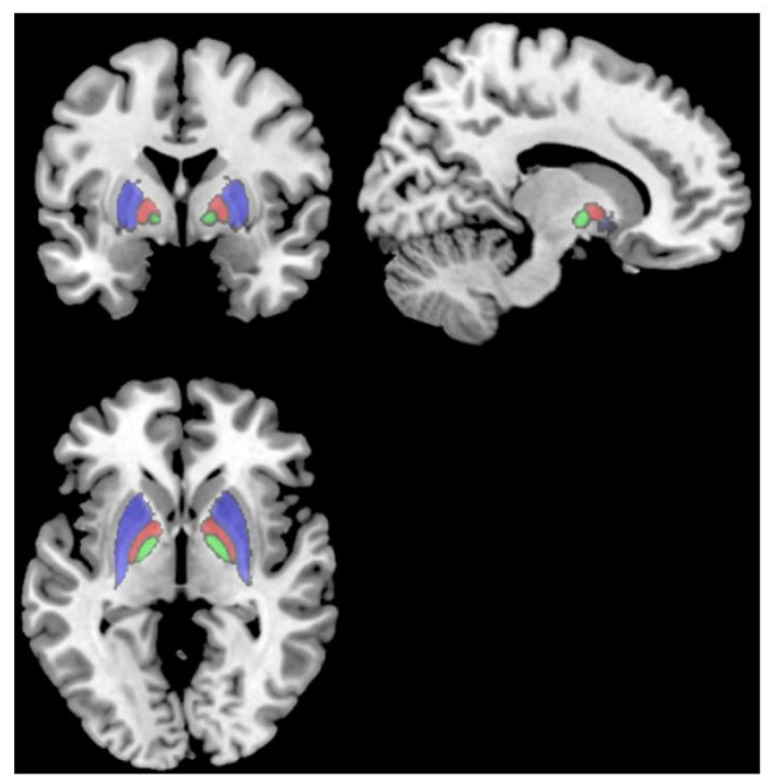
T1 MRI showing the putamen (red), pallidum (cyan), and thalamus (green), which all had smaller volumes in subjects > 6 months post-infection than subjects < 6 months post-infection [23].

Lastly, Deters et al. [23] explored brain volume and glucose metabolism changes in 33 people with prior mild COVID-19, divided into a <6-month post-infection group (n = 18) and a >6-month post-infection group (n = 15). The >6-month group showed smaller volumes in the putamen, pallidum, and thalamus compared to the <6-month group. Fatigued subjects in the >6-month group also had smaller frontal lobe volumes than non-fatigued subjects. Worse fatigue severity and perceived fatigability were associated with smaller frontal lobe volumes in the >6-month group. There were no differences in brain glucose metabolism between the <6-month and >6-month groups. However, both groups showed hypo- and hypermetabolism in certain regions compared to a healthy normative database. The results suggest that mild COVID-19 may lead to delayed decreases in subcortical and frontal lobe volumes, particularly in those with persistent fatigue (Figure 2; [23]). The mechanisms are unclear but could involve basal ganglia–cortical circuits underlying motivation and motor control.

MRI and DTI analyses demonstrate abnormal changes in basal ganglia architecture during the early phase of long-COVID recovery. The mismatch between different connectivity pathways hints at a maladaptive rewiring process. Further exploration of how these lesions progress over time and correlate with symptoms could shed light on the mechanisms of long-term neurological sequalae and inform interventions.

As investigators continue elaborating on the underpinnings of long-COVID’s neurological impact, mounting neuroimaging evidence converges on basal ganglia dysregulation as a candidate substrate perpetuating fatigue and cognitive dysfunction. Moving forward, longitudinal characterization of metabolic, functional, and structural changes within cortico-basal ganglia circuitry holds promise for developing diagnostic biomarkers and rational therapeutic targets for burdensome long-haul symptoms.

## 3. Theories on Causative Mechanisms

There are three predominant interrelated theories describing potential pathways leading from observed basal ganglia abnormalities to the extreme fatigue plaguing long-COVID survivors.

### 3.1. Inflammation-Induced Dopamine Signaling Dysfunction

The basal ganglia rely critically on dopamine neurotransmission to drive motivation and effort generation [24,25,26]. Dopamine signals project from the substantia nigra to dorsal and ventral striatal regions, modulating neuronal excitability. These dopaminergic projections and their targets play central roles in encoding reward prediction errors and incentive salience—judging effort requirements against likely rewards to guide cost/benefit decision-making underlying motivated behavior [27,28,29,30]. Emerging PET studies reveal that long-COVID neuroinflammation localizes within multiple basal ganglia nuclei, including substantia nigra dopamine projection sites, as well as dopamine–recipient putamen and caudate regions [10]. Localized microglial activation could provoke excitotoxic imbalances in dopamine availability or release dynamics. Animal models demonstrate that substantia nigra inflammation disrupts nigrostriatal dopamine transmission through oxidative damage [20]. Cytokine exposure downregulates tyrosine hydroxylase and dopamine active transport in culture [30]. Similar mechanisms in long-COVID basal ganglia circuits could distort reward valuation and effort computation mediated by the striatum [27,28,29]. With dopamine signals failing to accurately translate expected pleasure into motivation for action, behavioral activation may falter. Rather than displaying overt motor deficits, more subtle erosion of willingness to exert effort when rewards seem inadequate could manifest as fatigue. This proposed cascade linking inflammatory basal ganglia changes to dopamine dysfunction and motivational impairments warrants further exploration as a candidate mechanism underlying long-COVID exhaustion. Clarifying the contributions of neuroinflammation versus excitotoxicity or transport changes to dopamine dysregulation could guide therapeutic development.

### 3.2. Disruption of Cortical-Striatal Motivational Pathways

The basal ganglia are strategically situated at the intersection of limbic, cognitive, and motor loops to integrate motivation with action [31]. Dopaminergic projections from the ventral tegmental area and substantia nigra provide value and salience signals to striatal areas that also receive contextual input from the hippocampus, amygdala, and prefrontal cortex [32]. The striatum processes these diverse streams to estimate effort requirements and likely rewards, shaping an impression of potential action value. Globus pallidus and substantia nigra reticulata nodes send integrated information on motivation and movement planning to the motor cortex directly and via thalamocortical circuits [31]. The resulting output to dorsolateral prefrontal areas closes the loop, linking action values to higher-order decisions about behavioral activation [33]. If inflammation or structural alterations disrupt communication between key basal ganglia nodes, this complex risk/benefit calculus could become noisy or biased. The impression of all potential actions requiring unacceptable efforts for paltry rewards manifests psychologically as fatigue or loss of motivation [34]. Maladaptive upregulation of TGF-beta signaling presents one candidate mechanism by which cytoarchitectural reorganization between the striatum, pallidum, and substantia nigra could reweight cortico-striatal pathways from effortful behaviors [35,36]. Testing whether anti-inflammatory agents forestall such network-level motivational deficits could clarify contributions to long-COVID fatigue.

### 3.3. Loss of Excitatory Basal Ganglia Input to Arousal Centers

In addition to guiding motivation, the basal ganglia also play an integral role in modulating sleep–wake transitions and allocating attentional resources to tasks requiring cognitive effort [37,38]. As part of regulating arousal and attention, excitatory projections extend from the substantia nigra, ventral pallidum, and ventral tegmental area to various nodes of the ascending arousal system. These include intralaminar and midline thalamic nuclei with diffuse cortical projections, as well as brainstem nuclei activating histaminergic, noradrenergic, serotonergic, orexinergic, and cholinergic systems [37]. Through these pathways, the basal ganglia provide a measure of stimulatory drive that serves to stabilize cognition and alertness. PET studies indicate long-COVID neuroinflammation within basal ganglia structures contributing to arousal regulation, including substantia nigra [10]. Animal models also demonstrate the selective loss of striatal dopamine neurons following infection [39]. The resulting attenuation of excitatory outputs to thalamic and brainstem targets could impair the ability to sustain task-oriented attention and wakefulness. With the basal ganglia no longer providing adequate stimulatory inputs to counter homeostatic sleep pressure, symptoms resembling chronic exhaustion or mental fog emerge [38].

Probing whether basal ganglia inflammation predicts subsequent arousal deficits in people with long-COVID could clarify the role of this proposed mechanism in their fatigue. Selective arousal-enhancing agents might also relieve symptoms by compensating for depleted excitatory signaling.

## 4. Fatigue-Related Symptoms and Basal Ganglia Dysfunction

The pandemic response measures like quarantining and social isolation, while effective at slowing viral spread, may unintentionally worsen fatigue in COVID-19 patients by increasing health issues like post-traumatic stress, anxiety, depression, and pain. These negative psychological consequences are thought to significantly contribute to fatigue levels [40].

Studies estimate that 67–80% of long haulers report fatigue, exceeding that of controls even at 12 months post-infection [41,42,43]. These patients frequently experience comorbid stress, anxiety, depression, and widespread pain. For example, more than half of those with long-COVID have clinical levels of anxiety and depression [3]. Pain symptoms also affect over half of patients, including headaches, nerve pain, muscle soreness, joint pain, and chest pain [44]. Researchers propose that long-COVID fatigue and pain may be driven by inflammatory factors triggering central sensitization [45,46]. Psychological distress in long haulers correlates significantly with greater fatigue, poorer sleep, and reduced quality of life, demonstrating interconnectivity [47]. As persistent symptoms like fatigue accumulate, they can trigger stress and mood issues. Multimodal approaches addressing inflammation, neural pathways, mental health, and pain holistically may improve fatigue and associated symptoms in long-COVID.

Emerging evidence suggests that dysfunction in the basal ganglia may contribute to persistent fatigue-related symptoms in long-COVID patients. The basal ganglia play an important role in effort generation and motivation, which are impaired in many experiencing severe fatigue. Researchers have found a buildup of toxic metabolic byproducts in the basal ganglia of long-COVID patients, which correlates with reduced motivation and decreased voluntary activation measured by MRI [48]. Dysfunction in basal ganglia communication circuits could diminish motivation to engage in activities requiring sustained effort. Altered motivation and effort generation mediated through basal ganglia pathology could also perpetuate other linked symptoms like depression, anxiety, and pain in long haulers by reducing engagement in productive behaviors. Disruptions between the basal ganglia, anterior cingulate cortex, and insula may further compound fatigue and pain symptoms in long-COVID [47]. Assessing and targeting basal ganglia dysfunction through motivational interventions or neuromodulatory techniques could provide therapeutic benefits for fatigue, pain, stress, and mood disturbances related to long-term COVID sequelae.

Strikingly, the complex multi-system profile of long-COVID bears a remarkable resemblance to several established chronic illnesses for which severe fatigue is also a hallmark. These include post-viral syndromes like myalgic encephalomyelitis/chronic fatigue syndrome (ME/CFS), as well as fibromyalgia, Gulf War Illness, and multiple sclerosis (MS) [48]. Across these disorders, emerging understanding points to common underlying disease mechanisms that may promote comparable fatigue-related illness trajectories. For example, anxiety and depression have been shown to predict fatigue severity in chronic illness populations [49,50]. Conversely, fatigue can exacerbate emotional distress and perceived stress levels [51,52]. Pain intensity and fatigue levels also influence each other, with greater pain predicting increased fatigue and greater fatigue amplifying pain symptoms [53,54]. Researchers have proposed shared underlying biological pathways, including hypothalamic–pituitary–adrenal (HPA) axis dysregulation, inflammation, oxidative stress, and central nervous system sensitization as drivers of this symptom cluster [50,55].

Adding to the evidence on shared mechanisms of persistent fatigue, emerging research also suggests that basal ganglia dysfunction may underlie motivational deficits and effort intolerance, crossing many disorders. The basal ganglia play an integral role in effort generation, reward processing, and motor control. In injuries or illnesses triggering chronic fatigue like TBI, Parkinson’s disease, and post-viral syndromes, neuroimaging reveals suppressed activation in basal ganglia structures during effort-demanding tasks [56]. Researchers propose that dysfunction in nigrostriatal dopamine signaling reduces motivation to engage in behaviors requiring sustained effort, precipitating pervasive fatigue and related psychosocial withdrawal. Similar patterns of blunted striatal dopamine are observed in long-COVID, correlating with patient-reported effort intolerance and fatigue [12]. As in other fatigue conditions like ME/CFS, aberrant basal ganglia communication could diminish the willingness to expend the effort required for typical daily activities in long haulers. Treatments targeting increased dopamine availability may relieve effort-related exhaustion. Further interrogation of basal ganglia pathology provides another point of translational convergence across chronic fatigue landscapes.

Treatments targeting these mechanisms may have efficacy in addressing the multiple correlated symptoms. Given the interrelationships between fatigue, stress, mood disorders, and pain, assessment of these symptoms needs to be coordinated, and management should adopt a holistic approach.

## 5. Therapeutic Opportunities

As evidence crystallizing basal ganglia contributions to long-COVID mounted, researchers have piloted treatments aimed at addressing subcortical abnormalities underpinning fatigue, including the following.

Anti-inflammatory agents. Immunomodulatory medications like colchicine and intravenous immunoglobulin (IVIG) infusions could alleviate localized neuroinflammation centered on the basal ganglia documented in imaging studies [57]. In an open-label study, 14 long-COVID patients receiving 1 month of IVIG experienced marked and sustained improvements in fatigue as inflammation resolved on PET scans [58]. More rigorous evidence comes from a 3-month randomized trial finding that colchicine eased both cognitive dysfunction and fatigue versus placebo [57]. As a microtubule inhibitor used to treat gout, colchicine possesses anti-inflammatory properties that appear beneficial in long-COVID. Specifically, by suppressing microglial activation and attendant release of cytokines, like IL-1β, TNF-α, and nitric oxide, colchicine may interrupt self-amplifying cycles of immune-mediated dopaminergic injury centered on the basal ganglia [59,60]. Researchers posit that subsequent reductions in aberrant microglial pruning allow recovery of disrupted motivation/reward and motor control pathways [60].

Open questions remain regarding optimal agents, timing, and duration of immunomodulation for long COVID fatigue and cognitive symptoms. Nevertheless, early successes provide grounds for cautious optimism. Targeting neuroinflammation and its secondary impacts on subcortical structures may ameliorate the activity intolerance that is so incapacitating for long haulers working to rebuild their lives post-COVID.

Dopaminergic therapies. Strategies enhancing dopamine signaling via dopamine precursors or D2/D3 receptor agonists might counteract deficits within striatal motivation circuitry [61,62]. By amplifying decisional impulse-to-action conversion, dopaminergic treatments could conceivably energize initiation. Nevertheless, optimal dosing regimens remain uncertain, given the possible downregulation of dopamine receptors with chronic stimulant exposure [63]. More research should explore combined approaches, like pairing immunomodulators that resolve neuroinflammation with dopaminergic agents that reboot intrinsic reward circuity damaged secondarily. Early successes justify optimism that pharmacological strategies targeting the dopamine deficiency underpinning long-COVID fatigue can help patients reclaim their lives. Still, we must utilize such treatments judiciously until larger trials clarify ideal substance options, durations, and risk profiles.

Neurostimulation. Non-invasive stimulation using electromagnetic or ultrasonic pulses to extinguish inflammation foci in the basal ganglia holds promise for long-COVID [64]. Similarly, modalities like tDCS aimed at recalibrating cortico-striatal connectivity through electrical modulation could rebalance pathways, maintaining effortful engagement [65].

Noda et al. [66] demonstrated that repetitive transcranial magnetic stimulation (rTMS) can significantly improve fatigue and cognitive dysfunction in long-COVID when applied to the left dorsolateral prefrontal cortex (DLPFC) in 20 daily sessions [4]. Researchers posit that stimulating connected subcortical regions may help rebalance disrupted neurotransmission between inflammation-damaged nuclei.

Other groups have piloted implantable electrodes targeting basal ganglia structures, finding marked improvements in fatigue following high-frequency deep brain stimulation of the nucleus accumbens in five long haulers [67]. Though an invasive last resort, preliminary successes argue such neuromodulation could help reboot dopamine deficiency syndromes when medication and counseling fail [68].

Clearly, further research should optimize stimulation parameters and patient selection, given the variability in long-COVID neurological manifestations. However, early findings provide grounds for hope that directly altering the activity of fatigue-related brain circuitry could help long haulers reclaim their lives.

Multimodal approaches. Likely needed are combinations integrating anti-inflammatory and pro-dopaminergic medications and connectome-guided neurostimulation to holistically restore basal ganglia form and function [15]. This “network optimization” strategy accounts for the interdependence of structure, neurochemistry, and communication flow.

## 6. The Challenges of Long-COVID Fatigue Research

It should be noted that long-COVID fatigue research faces several critical challenges that have constrained progress in understanding its mechanisms, developing biomarkers, and advancing effective treatments. Defining and objectively quantifying fatigue itself poses barriers. As a subjective, multifaceted sensation relying heavily on self-report, fatigue evades precise characterization [69]. Measuring tools also remain inconsistent, with little consensus on the optimal scales despite options like the Fatigue Severity Scale and 11-item Chalder Fatigue Scale. Determining the etiology behind persisting exhaustion further complicates long-COVID investigations. Whether fatigue stems directly from the biological impacts of SARS-CoV-2 infection or links more to psychosomatic factors and patients’ beliefs about having had COVID-19 is unclear [69]. Plus, comparable objective fatigability between long-COVID patients with versus without fatigue suggests discrepancies between perceived versus measurable manifestations [70]. Such complex associations between psychology, physiology, and performance obscure causal attributions. Differentiating between perceived and objective aspects of fatigue, developing specific measurement tools, and elucidating potential psychosomatic contributors and neural correlates could progress research toward much needed treatment breakthroughs.

Emerging artificial intelligence (AI) methods, including machine learning and deep learning, show promise for consolidating personalized multi-omics data, behavior, and neural signals to advance fatigue research [71]. These techniques could help integrate profiles spanning genetics, molecular markers, self-reports, neuroimaging, and task performance to characterize the heterogeneity in how fatigue manifests across individuals. By creatively applying predictive analytics and data mining to such multifaceted datasets, researchers may overcome long-standing obstacles related to capturing individual variability in fatigue experiences. Additionally, machine learning provides approaches to glean insights from complex indicators without relying solely on invasive procedures for measurement. Progress will rely on effective communication and partnerships across disciplines to compile robust consolidated data resources that fully harness these versatile AI capacities for pattern recognition within fatigue’s biological complexity [71].

## 7. Conclusions and Future Directions

While neuroinflammation is a known phenomenon following viral and systemic illnesses, this narrative review sheds new light on the specific impact of long-COVID on the basal ganglia and its unique consequences. The review highlights the distinctive pattern of neuroinflammation, metabolic disruption, and structural alterations within the basal ganglia that sets long-COVID apart from other post-viral syndromes. The localization of these changes in the striatal dopamine circuitry and their association with motivational deficits and fatigue severity suggest a novel pathophysiological mechanism underlying long-COVID’s persistent neurological effects.

Moreover, the review emphasizes the potential role of the basal ganglia in integrating diverse aspects of long-COVID symptomatology, including cognitive impairment, emotional disturbances, and motor control issues. This multifaceted involvement of the basal ganglia in long-COVID is a unique feature that distinguishes it from other viral illnesses, which may have more limited or diffuse neurological impacts.

Another novel aspect of this review is the discussion of emerging therapeutic interventions targeting the basal ganglia, such as anti-inflammatory agents, dopaminergic medications, and neuromodulation techniques. These targeted approaches, informed by the specific basal ganglia abnormalities observed in long-COVID, represent a promising avenue for managing the persistent neurological sequelae of this condition.

However, the review also highlights significant gaps in understanding the precise mechanisms linking basal ganglia dysfunction to long-COVID fatigue, establishing causality and validating treatment efficacy. These gaps underscore the need for further research to elucidate the unique pathways through which long-COVID affects the basal ganglia and to develop targeted interventions that can effectively mitigate its neurological consequences.

In conclusion, this narrative review provides new insights into the distinctive impact of long-COVID on the basal ganglia, its multifaceted consequences, and potential therapeutic targets. While neuroinflammation is a shared feature of many viral and systemic illnesses, the specific pattern of basal ganglia involvement in long-COVID sets it apart and underscores the need for tailored interventions. Advancing our understanding of these unique subcortical drivers could profoundly improve the quality of life for the growing population affected by long-COVID neurological sequelae in the years to come.

## Figures and Tables

**Table 1:** Key findings related to basal ganglia changes and their associations with long-COVID fatigue and other persistent symptoms across multiple studies.

Reference	Subjects	Disease Duration	Comorbidities	Hospitalization	Observed Brain Changes
Douaud et al., [10]	600+ SARS-CoV-2 infected	Not specified	Cognitive impairment	Not specified	Connectivity weakening between basal ganglia, thalami, and sensory regions
Lu et al., [12]	Long-COVID patients vs. controls	10 months	Hypertension, memory loss, headache, tremor, impaired mobility, myalgia	Hospitalized	Striatal neuroinflammation compromised neuronal integrity in caudate and putamen
Kandemirli et al., [14]	ICU COVID-19 patients	Not specified	Hypertension, diabetes mellitus, cerebrovascular accident, chronic kidney disease, coronary artery disease	In ICU	56% had basal ganglia hyperintensities, reduced NAA/Cr and NAA/Cho ratios
Helms et al., [13]	58 Severe COVID-19 patients	Not specified	Confusion, cognitive dysfunction	Hospitalized	Basal ganglia hyperintensities (MRI)
Hampshire et al., [18]	Recovered COVID-19 patients	Not specified	Anxiety, depression, lung conditions, psychiatric conditions	Hospitalized	Cognitive deficits linked to disrupted basal ganglia communication
Meinhardt et al., [19]	33 deceased COVID-19 patients	Not specified	Not specified	Not specified	Olfactory SARS-CoV-2 invasion as path to central nervous system
Zhou et al., [20]	Recovered COVID-19 patients	Nearly 1 year	Diabetes, hypertension, hyperlipidemia	Hospitalized 17.5–41.5 days	Reduced white matter integrity in nigrostriatal pathways
Hafiz et al., [21]	46 COVID-19 vs. 30 controls	2 weeks post-discharge	Fatigue	Hospitalized	Higher gray matter volume in limbic regions and basal ganglia; correlation with fatigue
Heine et al., [22]	50 long-COVID vs. 47 controls	Median 7.5 months	Anxiety, depression, sleep problems in long-COVID	13% Hospitalized	Thalamus and basal ganglia volume loss, surface deformations, altered diffusion; correlated with fatigue
Deters et al., [23]	33 mild COVID-19 patients	<6 months (*n* = 18) vs. >6 months (*n* = 15)	Persistent fatigue in some >6 month patients	Not hospitalized	Smaller putamen, pallidum, thalamus volumes in >6 month group, especially fatigued; frontal hypometabolism
Luo et al., [17]	32 post-COVID-19 patients (16 fatigued, 16 non-fatigued)	6.9 ± 4.8 months (FT), 8.5 ± 5.7 months (NF)	Not specified	Not hospitalized	Decreased globus pallidus activity in both fatigued and non-fatigued groups compared to healthy controls; non-fatigued group showed greater hypometabolism; right hemisphere more affected in both groups
